# The Effectiveness of Cryoflow Cooling on Forearm Skin Temperature and Nerve Conduction Velocity in Normal Subjects: A Case–Control Study

**DOI:** 10.3390/neurosci7010001

**Published:** 2025-12-24

**Authors:** Mohamed Salaheldien Alayat, Kadrya H. Battecha, Yazeed Saleh Jabr, Faisal Zagzoog, Baraa Hasaballah, Faisal Faleh Saud Alsulami, Matuq Abdullah Refaei, Osama Saleh Almehmadi

**Affiliations:** Medical Rehabilitation Science Department, College of Applied Medical Science, Umm Al-Qura University, Makkah 21421, Saudi Arabia; khbattecha@uqu.edu.sa (K.H.B.); s443004210@uqu.edu.sa (Y.S.J.); s442013081@uqu.edu.sa (F.Z.); s443004836@uqu.edu.sa (B.H.); s443003154@uqu.edu.sa (F.F.S.A.); s443007812@uqu.edu.sa (M.A.R.); s443009006@uqu.edu.sa (O.S.A.)

**Keywords:** Cryoflow cryotherapy, local skin temperature, infrared camera, motor and sensory nerve conduction velocity

## Abstract

**Objectives**: The aim of this study was to investigate the effectiveness of Cryoflow cooling on forearm skin temperature and nerve conduction velocity (NCV) in normal subjects. **Methods**: Thirty male volunteers participated in this study, with a mean age of 20.8 ± 0.74 years. A Cryoflow hose with a nozzle was positioned approximately 10 cm from the forearm and scanned the anterior surface of the non-dominant forearm for 10 min, with temperatures adjusted to −10 °C. Participants’ average skin temperature was measured by using an infrared camera. Motor and sensory NCV for both the median and ulnar nerves were measured from both forearms. The dominant side served as a control side. The level of significance was set at *p* value ≤ 0.05. **Results**: Following treatment, the experimental group experienced a reduction in average skin temperature, dropping from 32.94 ± 1.11 °C to 16.92 ± 1.68 °C, while the control group showed no significant change. Both the median and ulnar nerves exhibited significant decreases in motor NCV (−10.37 m/s and −8.79 m/s, respectively), alongside slight increases in distal motor latency. Sensory NCV of the median and ulnar nerves decreased significantly (−5.20 m/s and −8.40 m/s, respectively), accompanied by increased onset latency. No significant changes were found in the control group. **Conclusions**: Cryoflow air-based cryotherapy to the forearm causes a substantial reduction in local skin temperature and significant slowing of peripheral nerve conduction. Both motor and sensory fibers of the median and ulnar nerves exhibited decreased conduction velocities and increased latencies following cooling.

## 1. Introduction

Cryotherapy refers to the therapeutic use of cold to alleviate various medical and musculoskeletal conditions [1]. It is one of the most frequently applied modalities in physical therapy, sports medicine, and rehabilitation practice [2] and is commonly administered during both the acute and recovery phases of injury management [3]. For decades, cryotherapy has been recognized as an effective, low-cost, and straightforward intervention for controlling pain and inflammation following acute sports-related trauma [4].

Different cooling methods have been utilized in clinical and athletic settings, including crushed ice packs, ice massage, and cold water immersion, each varying in cooling efficiency. The degree of temperature reduction achieved depends largely on the cooling modality, treatment duration, the surface area exposed, and the individual’s activity level before and after treatment [5]. In general, the therapeutic effects of cold application include decreased pain, reduced muscle spasm, and the prevention of post-traumatic edema through lowering tissue metabolism and nerve conduction velocity (NCV) [6,7].

When cold is applied to the body, local skin temperature declines rapidly during the first few minutes of exposure. The magnitude of cooling depends on factors such as modality type, application time, and the anatomical characteristics of the target region. Previous studies reported that skin temperature may reach 10–15 °C after 15–20 min of cryotherapy, which is sufficient to trigger vasoconstriction, reduce enzymatic activity, and produce analgesic effects [8]. These physiological responses collectively contribute to inflammation control and pain reduction. Several investigations have compared the efficiency of common cryotherapy techniques, identifying crushed ice packs, ice massage, and cold water immersion as the most effective methods for reducing superficial tissue temperature [9].

Electrophysiological measures such as NCV and electromyography are well-established tools for assessing peripheral nerve function in both healthy individuals and patients with neurological disorders [10]. NCV testing evaluates the ability of motor and sensory fibers to transmit electrical impulses and is a standard component of neurophysiological assessment [11]. The precision of NCV measurements relies on consistent electrode placement and temperature control, as conduction speed is highly temperature-dependent. A decrease in limb temperature can substantially slow nerve conduction, while elevated temperature accelerates it.

Previous studies have confirmed that different cryotherapy modalities influence NCV in varying degrees. For example, a recent investigation comparing ice packs, ice massage, and cold water immersion on sural and posterior tibial nerves found significant reductions in NCV across all modalities, with the sensory fibers showing greater sensitivity to cooling. Among these methods, cold water immersion achieved the greatest decrease in NCV, likely due to its larger cooling area [12]. Another report observed that a drop in ankle skin temperature was directly associated with a decline in NCV [13]. Conversely, cases of transient nerve palsy have been described following ice application near superficial nerves such as the ulnar nerve at the elbow [14].

Several factors account for these variable outcomes, including the type and duration of the cooling method, as well as the anatomical depth of the targeted nerve. Peripheral nerves located close to the skin surface (e.g., ulnar or peroneal nerves) are more affected by cooling compared to deeper structures like the sciatic nerve [12,15]. Consequently, cryotherapy protocols should be individualized according to the site, duration, and intensity of application to optimize clinical benefits and reduce potential complications such as temporary conduction block or cold-induced neuropathy.

Cryoflow therapy represents a relatively new, contact-free cryotherapy modality that delivers a continuous stream of air cooled to subzero temperatures, guided by infrared temperature sensors. This technology minimizes frostbite risk, provides uniform cooling, and allows for dynamic scanning of the treatment area for improved comfort and precision. However, despite its clinical adoption, little is known about the physiological impact of Cryoflow on peripheral nerve function. To date, no published studies have objectively quantified the effects of Cryoflow air-based cryotherapy on local skin temperature or peripheral nerve conduction parameters. Cooling-induced alterations in nerve conduction have been extensively documented, with reductions in nerve temperature shown to decrease conduction velocity, prolong latency, and increase temporal dispersion in both motor and sensory fibers. Experimental and clinical studies have demonstrated a predictable relationship between temperature reduction and NCV slowing, typically in the range of approximately 2–2.5 m/s per 1 °C decrease in nerve temperature. These findings provide a well-established neurophysiological basis for the use of cryotherapy and inform the rationale for examining the effects of newer air-based cooling technologies. Furthermore, the existing literature primarily focuses on traditional cryotherapy methods, leaving a gap regarding this modern air-cooling approach. Therefore, the present study aimed to investigate the effectiveness of Cryoflow cooling on forearm local skin temperature and on motor and sensory nerve conduction velocity of the median and ulnar nerves in healthy adults.

## 2. Materials and Methods

### 2.1. Study Design

This case–control study explored the influence of localized Cryoflow cooling on forearm skin temperature and peripheral nerve conduction in healthy adults. Ethical approval was obtained from the Biomedical Research Ethics Committee at Umm Al-Qura University, Makkah, Saudi Arabia (approval no. HAPO-02-K-012-2025-01-1276). Participants were recruited from the Physical Therapy Program, Department of Medical Rehabilitation Sciences, College of Applied Medical Sciences, Umm Al-Qura University.

### 2.2. Participants

The required sample size was calculated using G-Power software (version 3.1, Heinrich-Heine University, Düsseldorf, Germany). Based on an estimated effect size of 0.8, α = 0.05, and statistical power of 0.95 for matched-pair comparisons, a minimum of 23 participants was required. To compensate for potential dropouts, the sample size was increased to 30. Thirty healthy male volunteers (aged 20–23 years) were randomly selected from the student population. The effect size (Cohen’s d = 0.8) was selected based on previous cryotherapy studies reporting large within-subject changes in skin temperature and nerve conduction velocity following local cooling interventions [12,13]. All participants gave written informed consent before inclusion. Eligibility criteria included the following: (1) normal neurological examination, (2) the absence of upper-limb pain or weakness, and (3) right-hand dominance. (4) Participants were instructed to refrain from strenuous physical activity, caffeine, and alcohol consumption for at least 12 h prior to testing to minimize factors that could influence baseline skin temperature. Exclusion criteria included any history of upper-limb injury, neurological disorder, peripheral neuropathy, metabolic disease (e.g., diabetes or thyroid disorders), use of medications affecting neuromuscular function, or abnormal findings on neurological examination, as these factors are known to influence skin temperature regulation and NCV. The study sample consisted of 30 healthy male participants aged 20–23 years. Anthropometric characteristics, including height, body mass, body mass index (BMI), and forearm length, were recorded to characterize the study population and to account for factors known to influence skin temperature and nerve conduction parameters.

#### 2.2.1. Measurement of Local Skin Temperature

Forearm surface temperature was assessed using a non-contact infrared thermal imaging camera (FLK-TIS10, Fluke^®^, Everett, WA, USA). This imaging technique provides reliable, high-resolution surface temperature data. To ensure consistency, all measurements were performed in a climate-controlled laboratory (22 °C, 60% relative humidity) with minimal air flow. Participants acclimatized for 15 min before data collection [16,17,18]. Prior to data collection, the thermal imaging camera (FLK-TIS10, Fluke^®^, Everett, WA, USA) was calibrated according to the manufacturer’s recommendations. Calibration was verified using an internal automatic calibration routine before measurements. Emissivity was set at 0.98, which is the standard value for human skin, and reflected ambient temperature was entered according to room conditions. All measurements were performed under standardized conditions to ensure measurement accuracy and repeatability. Infrared thermography has been shown to provide reliable and valid measurements of human skin temperature when such calibration and environmental controls are applied [18,19].

Each participant lay in a supine position with both forearms supported at heart level and palms facing upward. The camera was positioned 1 m from the volar aspect of the forearm. Thermal images of both forearms (experimental and control) were captured before and immediately after the cooling intervention, with the left forearm designated as the treatment side. Thermal images were analyzed using the accompanying software (SmartView 4.3 for Windows, Fluke^®^, Everett, WA, USA). The region of interest (ROI) was standardized for all subjects, and average temperature within the ROI was automatically computed. This method allowed for precise quantification of temperature variations induced by Cryoflow treatment. Measurement procedures were standardized and performed by the same examiner to minimize measurement variability.

#### 2.2.2. Nerve Conduction Velocity Assessment

Motor and sensory NCV studies were performed for both the median and ulnar nerves using a standard electromyography and nerve conduction system (Cadwell^®^ Sierra^®^ Summit™, Kennewick, WA, USA). The laboratory temperature was maintained at 22 °C to ensure physiological stability during testing. Prior to electrode placement, the skin was cleansed with alcohol to minimize impedance.

For the median nerve motor study, the active electrode was positioned over the abductor pollicis brevis (APB) muscle and the reference electrode over the distal phalanx of the thumb. Stimulation was applied at two points: (1) the proximal wrist crease (approximately 7–8 cm from the APB) and (2) the antecubital fossa, medial to the biceps tendon. Motor conduction velocity was calculated by dividing the distance between stimulation sites by the latency difference.

For the ulnar nerve motor study, the recording electrode was placed over the abductor digiti minimi (ADM) muscle and the reference on the fifth digit. Stimulation was delivered at the wrist (7–8 cm proximal to the ADM) and at the elbow, just distal to the ulnar groove [19,20]. The distal latency, amplitude of the compound muscle action potential (CMAP), and conduction velocity were recorded.

Sensory conduction studies were performed using the orthodromic technique. For the median nerve, stimulation was applied to the index finger (digit II) via ring electrodes, and recording electrodes were positioned over the median nerve at the wrist (between palmaris longus and flexor carpi radialis tendons), about 12–14 cm proximal to the stimulation site.

For the ulnar nerve, stimulation was delivered to the little finger (digit V) with recordings taken at the wrist, lateral to the flexor carpi ulnaris tendon. Electrical stimuli consisted of 0.1–0.2 ms square-wave pulses at supramaximal intensity. Measurements were collected from both sides before and immediately after the cooling procedure. Measurement procedures were standardized and performed by the same examiner to minimize measurement variability. All nerve conduction procedures were performed in accordance with standard clinical electrodiagnostic guidelines and established methodologies for peripheral nerve assessment [20,21].

### 2.3. Cryotherapy Protocol

Cryotherapy was administered using the Cryoflow^®^ system (Gymna, Bilzen, Belgium), which delivers a continuous stream of air cooled to approximately −10 °C. Participants were seated with their forearm resting comfortably and fully exposed, palm facing upward. The Cryoflow hose and nozzle were positioned about 10 cm above the volar forearm and moved continuously to maintain uniform cooling. The cooling session lasted 10 min, with real-time temperature feedback provided by the integrated infrared sensor to ensure safe and consistent application. The participants’ contralateral forearm served as an untreated control. The Cryoflow cooling procedure was standardized and performed by the same therapist to minimize intervention variability.

### 2.4. Statistical Analysis

Data were analyzed using SPSS software (version 26, IBM Corp., Armonk, NY, USA). Descriptive statistics summarized participant demographics and baseline measures. Paired *t*-tests were used to compare pre- and post-intervention values within each group, while independent *t*-tests compared post-treatment differences between the experimental and control sides. Statistical significance was set at *p* ≤ 0.05, and all data are reported as the mean ± standard deviation (SD).

## 3. Results

A total of 30 healthy male participants completed this study. The mean (±SD) age was 20.8 ± 0.74 years, mean height was 173.07 ± 7.33 cm, mean weight was 78.73 ± 21.83 kg, mean forearm length was 27.48 ± 1.5 cm, and mean body mass index (BMI) was 26.23 ± 6.9 kg/m^2^. The Shapiro–Wilk test indicated no significant deviation from normal distribution (*p* > 0.05), confirming the suitability of parametric analyses.

### 3.1. Changes in Local Skin Temperature

Following the Cryoflow intervention, a marked decrease in forearm skin temperature was observed on the experimental side compared to baseline. The mean temperature dropped from 32.94 ± 1.11 °C before cooling to 16.92 ± 1.68 °C post treatment (*p* = 0.001). In contrast, the control forearm showed minimal change (33.31 ± 0.92 °C before vs. 33.44 ± 0.92 °C after; *p* > 0.05). Between-group comparison confirmed a highly significant difference in post-treatment temperature values (*p* ≤ 0.05). The average decline of 16.02 °C in the treated arm reflects the strong superficial cooling capacity of Cryoflow (Table 1 and Table 2).

### 3.2. Motor Nerve Conduction Parameters

In the experimental group, cooling significantly prolonged distal motor latency (DML) and reduced conduction velocity in both the median and ulnar nerves. For both median and ulnar nerves, there was a significant increase in DML with a significant decline in the motor NCV (Table 1 and Table 2). No significant differences were noted in the control forearm for either nerve. Intergroup comparisons of post-treatment values showed statistically significant differences in all motor parameters (*p* = 0.001). Overall, the median and ulnar motor NCVs decreased by 10.37 m/s and 8.79 m/s, respectively, reflecting the substantial effect of local cooling on motor fiber excitability (Table 1 and Table 2).

### 3.3. Sensory Nerve Conduction Parameters

Similarly to motor findings, sensory conduction velocities of both nerves were significantly reduced after cryotherapy. For the median nerve, onset latency increased, accompanied by a decrease in sensory NCV. For the ulnar nerve, onset latency changed while sensory NCV significantly dropped from the baseline value (*p* = 0.001). No significant variations were detected in the control side. Post-treatment comparisons between groups also revealed highly significant differences (*p* = 0.001). The mean reductions in sensory NCV were −5.20 m/s for the median nerve and −8.40 m/s for the ulnar nerve, indicating that sensory fibers were also substantially affected by cooling (Table 1 and Table 2).

## 4. Discussion

The present study demonstrates that localized Cryoflow cooling applied to the forearm produced significant physiological alterations in skin temperature and peripheral nerve conduction parameters without inducing abnormal responses or adverse effects. Compared with the control side, the treated forearm showed a marked drop in surface temperature and a notable reduction in both motor and sensory conduction velocities of the median and ulnar nerves. These findings confirm that Cryoflow effectively induces superficial cooling and transiently modulates nerve excitability.

### 4.1. Rationale for Studying Cryotherapy in Healthy Subjects

In the present study, Cryoflow cooling reduced forearm skin temperature to approximately 16.9 °C, representing a substantial decrease from baseline and sufficient to induce marked reductions in both motor and sensory NCV. Previous studies have suggested that skin temperatures in the range of 10–15 °C are commonly associated with pronounced vasoconstriction, enzymatic suppression, and analgesic effects [8]. These physiological responses are not governed by rigid temperature thresholds but instead occur along a continuum. Experimental evidence indicates that nerve conduction slowing and changes in axonal excitability begin at higher skin temperatures and progress with further cooling, supporting the interpretation that meaningful neurophysiological effects were achieved despite skin temperature remaining slightly above the traditionally cited range. While extending treatment duration may further reduce skin temperature, deeper or prolonged cooling over superficial nerves may increase the risk of excessive conduction slowing or transient neuropraxia. Therefore, the observed temperature reduction likely reflects a balance between achieving desirable physiological effects and maintaining safety, particularly for air-based cryotherapy applied to regions containing superficial neural structures.

Although this study was conducted in healthy individuals, this design aligns with established cryotherapy and neurophysiology research that uses normal subjects to define baseline physiological responses to cooling. Studying healthy populations allows for the isolation of the direct effects of temperature reduction on peripheral nerve function, minimizing confounding influences such as inflammation, edema, neuropathy, altered tissue perfusion, or medication effects commonly present in clinical populations. Experimental evidence indicates that temperature alone can account for substantial reductions in motor and sensory nerve conduction velocity, independent of disease processes [22,23]. This controlled approach is particularly important when evaluating newer cryotherapy technologies, such as air-based cooling systems, whose neurophysiological effects are not yet well characterized.

Accordingly, the present findings should be interpreted as reference physiological responses rather than direct indicators of clinical efficacy. In healthy neural tissue, the absence of pathological alterations allows for changes in nerve conduction velocity to be attributed solely to temperature effects. Previous studies have demonstrated predictable and reversible relationships between cooling, conduction slowing, and latency prolongation, with NCV declining by approximately 2–2.5 m/s for each 1 °C reduction in limb temperature [24,25,26]. The magnitude of NCV reduction observed in the current study closely matches these established relationships, supporting the physiological validity of Cryoflow-induced effects and providing essential reference data for comparison with clinical populations, where baseline nerve function is often impaired.

Data from healthy subjects also offer a mechanistic framework for clinical translation. In pathological conditions, baseline NCV is frequently reduced, and additional cooling may result in greater absolute conduction slowing or delayed recovery [11,26]. Quantifying NCV changes in normal tissue therefore allows clinicians to anticipate the potential magnitude and safety implications of cryotherapy responses in patients, particularly when applied over superficial nerves, where excessive cooling may transiently impair sensory or motor function [14].

Finally, healthy-subject studies are fundamental for validating cryotherapy modalities, establishing exposure parameters, and defining dose–response relationships prior to clinical use. Previous comparisons of conventional cryotherapy techniques have relied on healthy participants to characterize cooling efficiency and neural effects [12,13]. Extending this approach, the present study demonstrates that Cryoflow air-based cooling produces clinically relevant and reversible reductions in skin temperature and nerve conduction without adverse effects. These findings support its controlled clinical application and contribute to evidence-based guidance regarding safe cryotherapy use.

In clinical conditions where cryotherapy is commonly applied—such as acute musculoskeletal injury, postoperative inflammation, or tendinopathy—baseline skin temperature is frequently elevated due to increased local blood flow and inflammatory activity. Infrared thermography studies have reported skin temperature elevations of approximately 1–3 °C in injured or inflamed tissues compared with contralateral or healthy sites [8,17]. Consequently, the reduction in skin temperature observed in healthy participants in the present study may represent a conservative estimate of the cooling effect achievable in clinical populations, where larger thermal gradients may exist.

The observed decrease of approximately 16 °C following Cryoflow application falls within the range reported for effective analgesic cryotherapy and is sufficient to trigger vasoconstriction, reduce local metabolic activity, and alter neural excitability [12,13]. The accompanying slowing of nerve conduction observed in this study supports the established relationship between temperature and axonal function. Lower temperatures are known to delay depolarization by reducing sodium channel activation and prolonging their inactivation phase, resulting in decreased nerve conduction velocity and increased latency [25,26,27,28]. These mechanisms underpin the widespread clinical use of cryotherapy for pain modulation and inflammation control.

Consistent with this approach, the physiological effects of physical therapy modalities have been extensively examined in healthy individuals to characterize their thermal and biological responses prior to clinical application. Previous work demonstrated that infrared radiation produced a significant increase in local skin temperature, whereas electrical stimulation resulted in a non-significant thermal response [29]. Similarly, therapeutic ultrasound reported notable increases in skin temperature and skeletal muscle blood flow when applied at 3 MHz and an intensity of 2 W/cm^2^ in healthy participants [30,31]. Recently, a sustained rise in local skin temperature lasting up to 45 min was observed following a single session of thermal pulsed radiofrequency therapy in healthy subjects, reinforcing the importance of normal-subject models for evaluating the thermal characteristics and safety profiles of emerging physical therapy modalities [32]. More recently, studies have investigated the thermal effect of advanced laser technologies in normal participants [16,33]. Although laser therapy has traditionally been regarded as an athermal modality, these investigations demonstrated that high-intensity laser application can produce significant thermal effects when applied to the thigh and forearm regions of healthy subjects [16,33].

### 4.2. Effects of Cryoflow on Nerves in Healthy Versus Clinical Populations

Peripheral nerve conduction parameters differ substantially between healthy individuals and patients with neurological or musculoskeletal conditions. In healthy adults, upper-limb motor and sensory NCV values typically range between 50 and 65 m/s, whereas patients with nerve compression, peripheral neuropathy, or post-traumatic nerve irritation often demonstrate reduced baseline conduction velocities and prolonged latencies [11,26]. Experimental cooling has been shown to further slow NCV in both normal and diseased nerves, although the absolute magnitude of slowing may be greater in compromised neural tissue.

These findings are consistent with previous electrophysiological studies demonstrating that cooling-induced reductions in nerve temperature lead to decreased conduction velocity, increased latency, and greater temporal dispersion of action potentials in both motor and sensory fibers [12,34]. Experimental and clinical data indicate that nerve conduction velocity decreases by approximately 2 m/s for each 1 °C reduction in nerve temperature, supporting the magnitude of conduction slowing observed in the present study.

Both motor and sensory fibers exhibited significant decreases in conduction velocity following cryotherapy, though sensory fibers appeared slightly more affected. The median and ulnar motor NCV decreased by 10.37 m/s and 8.79 m/s, respectively, while the sensory NCV dropped by 5.20 m/s and 8.40 m/s. These results are consistent with previous studies that demonstrated cooling-induced slowing of both sensory and motor transmission [12,13,35,36]. The higher sensitivity of sensory fibers may be attributed to their smaller diameter and more superficial course compared to motor axons, making them more susceptible to thermal gradients.

A well-known linear relationship exists between nerve conduction velocity and temperature, with an estimated decrease of 2–2.5 m/s for every 1 °C drop in surface temperature [24,25]. The magnitude of reduction observed in this study aligns closely with that predicted rate, further validating the physiological plausibility of the results. Therefore, the reductions in NCV documented in the present study provide important mechanistic insight into the degree of conduction slowing that may occur during cryotherapy in clinical settings. In patients with heightened nociceptive input, temporary slowing of sensory conduction may contribute to analgesic effects, whereas excessive or prolonged cooling over superficial nerves could transiently impair motor or sensory function. Understanding these physiological responses in healthy tissue is essential for anticipating both therapeutic benefits and potential risks in patient populations.

### 4.3. Comparison with Traditional Cryotherapy Modalities

The observed Cryoflow-induced changes in NCV are comparable to those reported for traditional cryotherapy techniques, including ice packs, ice massage, and cold water immersion [12,13]. Previous studies have demonstrated that cold water immersion produces the greatest and most sustained reductions in NCV due to extensive tissue contact, whereas localized modalities result in more moderate but clinically meaningful effects. Despite being contact-free, Cryoflow achieved a magnitude of temperature reduction and conduction slowing similar to contact-based methods, suggesting that convective air-cooling can be an effective alternative modality.

Importantly, no adverse neurological symptoms were observed in this study. This contrasts with isolated reports of transient ulnar neuropathy following prolonged ice application over superficial nerves [14]. No adverse events were observed in the current study, likely due to the integrated infrared temperature feedback and continuous scanning mechanism of Cryoflow, which may reduce the risk of focal overcooling and support its safety when applied under controlled conditions.

The observed changes in NCV can be attributed to temperature-induced alterations in axonal membrane properties. Cooling decreases ion permeability and slows the kinetics of voltage-gated sodium channels, thereby extending depolarization duration and increasing the refractory period [25,26,27]. As a result, conduction velocity declines proportionally with temperature reduction until activity ceases at extreme cold levels. These effects are reversible upon rewarming and reflect temporary suppression of nerve excitability rather than structural injury [28]. The present findings are also supported by Mallette et al. [10], who observed a 30% reversible reduction in ulnar NCV accompanied by a 15% decrease in motor unit firing rate after localized forearm cooling. Such electrophysiological changes likely contribute to the analgesic and anti-spasmodic benefits of cryotherapy, as slower nerve transmission may dampen nociceptive signaling and muscle reflex activity [10].

Earlier reports have indicated that cold exposure causes predictable physiological shifts, with conduction velocity declining by approximately 5% for each 1 °C temperature drop [24,25,26]. Studies involving cold water immersion have shown that NCV and distal motor latency changes follow either linear or exponential trends over time [22,23,36,37]. The present results align with these observations and extend them to air-based cryotherapy, demonstrating comparable magnitudes of conduction slowing and latency prolongation. Herrera et al. [35] further noted that sensory NCV remains reduced for up to 30 min post cooling, particularly when participants remain at rest. This sustained suppression suggests that recovery of nerve excitability depends not only on rewarming but also on post-intervention activity levels. Although the current study measured immediate effects only, similar persistence could be expected after Cryoflow cooling.

The outcomes of this study reinforce the therapeutic rationale for using cryotherapy in pain and inflammation management. By transiently reducing NCV and metabolic activity, Cryoflow cooling can modulate peripheral excitability without the risks associated with contact-based modalities. Moreover, its infrared-guided airflow system allows for consistent cooling, improved patient comfort, and lower frostbite risk.

From a clinical perspective, the present findings provide a physiological framework for the safe and effective use of Cryoflow cooling. Cryoflow-induced cooling significantly reduces skin temperature and transiently slows both motor and sensory NCV in the forearm. Understanding the extent and duration of NCV reduction is important for anticipating physiological responses when cryotherapy is applied over regions containing superficial nerves. In particular, transient conduction slowing observed after cooling should be considered when interpreting or scheduling electrodiagnostic or neuromuscular evaluations following cold exposure. Transient reductions in NCV may contribute to analgesia and muscle relaxation, supporting the use of cryotherapy in acute injury and postoperative rehabilitation. However, clinicians should be aware that cooling over regions containing superficial nerves may temporarily alter sensory feedback or motor performance. These findings may assist clinicians in anticipating physiological responses to cryotherapy and interpreting nerve conduction measures following cooling, rather than implying therapeutic effectiveness. Future studies should extend these findings to clinical populations characterized by inflammation, pain, or nerve dysfunction, with particular attention paid to dose–response relationships, recovery time of NCV following cooling, and functional outcomes. Longitudinal designs incorporating follow-up measurements would further clarify the duration and clinical significance of Cryoflow-induced neural changes.

## 5. Conclusions

This study demonstrated that applying Cryoflow air-based cryotherapy to the forearm causes a substantial reduction in local skin temperature and significant slowing of peripheral nerve conduction. Both motor and sensory fibers of the median and ulnar nerves exhibited decreased conduction velocities and increased latencies following cooling, while no comparable changes occurred in the untreated control side.

These findings confirm that Cryoflow effectively modulates peripheral nerve excitability through temperature-dependent mechanisms. The capacity of this device to deliver precise, uniform cooling without direct skin contact highlights its clinical potential for managing pain, inflammation, and neuromuscular hyperactivity. By reducing nerve conduction velocity in a controlled and reversible manner, Cryoflow may serve as a safe and comfortable alternative to conventional contact-based cryotherapy methods.

Clinicians should, however, remain mindful of transient reductions in conduction speed when treating areas overlying major superficial nerves or when cryotherapy precedes electrophysiological testing. Overall, the results provide a physiological foundation for incorporating Cryoflow into therapeutic protocols aimed at modulating neuromuscular activity and pain perception.

## 6. Limitations

Although the present findings are promising, several methodological limitations should be acknowledged. The study sample consisted solely of young, healthy male participants, which restricts the generalizability of the results to other populations, including females, older adults, or individuals with neuropathic or metabolic disorders.

Additionally, the effects were measured immediately after cooling, without any follow-up assessments to determine the duration of conduction changes or the timeline of recovery. The current protocol employed only a single temperature setting and a 10 min exposure period. Exploring different intensities, durations, or air-flow rates could further clarify dose–response relationships. Moreover, the air-cooling mechanism primarily affects superficial tissues, so its influence on deeper neural structures remains uncertain. The present findings do not imply clinical efficacy but provide physiological context for future investigations in patient populations. Future studies incorporating post-intervention follow-up intervals would help establish how long Cryoflow-induced alterations in NCV persist.

Despite these limitations, this study provides valuable baseline data for future investigations and supports the feasibility of Cryoflow as a standardized, contact-free cooling modality for clinical and research applications.

## Figures and Tables

**Table 1:** Student’s *t*-test among the measured outcomes.

Variable	Interval	Experimental	Control	*p* Value
Average skin temperature (^°^C)	Pre-treatment	32.94 ± 1.11	33.31 ± 0.92	*p* > 0.05
	Post-treatment	16.92 ± 1.68	33.44 ± 0.92	0.001 *
	*p* value	0.001 *	*p* > 0.05	
Distal motor latency (Median) (ms)	Pre-treatment	3.81 ± 0.49	3.55 ± 0.30	*p* > 0.05
	Post-treatment	3.06 ± 0.15	3.52 ± 0.13	0.001 *
	*p* value	0.001 *	*p* > 0.05	
Motor NCV for median nerve (m/s)	Pre-treatment	61.03 ± 4.65	58.03 ± 2.52	*p* > 0.05
	Post-treatment	50.66 ± 5.38	58.36 ± 1.99	0.001 *
	*p* value	0.001 *	*p* > 0.05	
Distal motor latency (Ulnar) (ms)	Pre-treatment	3.91 ± 0.52	3.85 ± 0.30	*p* > 0.05
	Post-treatment	3.16 ± 0.52	3.82 ± 0.13	0.001 *
	*p* value	0.001 *	*p* > 0.05	
Motor NCV for ulnar nerve (m/s)	Pre-treatment	58.14 ± 5.21	57.20 ± 2.35	*p* > 0.05
	Post-treatment	49.35 ± 4.48	57.90 ± 2.55	0.001 *
	*p* value	0.001 *	*p* > 0.05	
Onset latency for median nerve (ms)	Pre-treatment	3.04 ± 1.12	3.87 ± 0.42	*p* > 0.05
	Post-treatment	2.83± 0.48	3.82 ± 0.22	0.001 *
	*p* value	0.001 *	*p* > 0.05	
Sensory NCV for median nerve (m/s)	Pre-treatment	55.06 ± 4.09	56.20 ± 2.24	*p* > 0.05
	Post-treatment	49.86 ± 3.01	56.90 ± 1.41	0.001 *
	*p* value	0.001 *	*p* > 0.05	
Onset latency for ulnar nerve (ms)	Pre-treatment	3.24 ± 1.25	3.34 ± 0.33	*p* > 0.05
	Post-treatment	2.71± 0.34	3.35 ± 0.12	0.001 *
	*p* value	0.001 *	*p* > 0.05	
Sensory NCV for ulnar nerve (m/s)	Pre-treatment	56.06 ± 3.19	56.18 ± 2.15	*p* > 0.05
	Post-treatment	47.66 ± 3.41	56.27 ± 1.62	0.001 *
	*p* value	0.001 *	*p* > 0.05	

*: Significant changes (Student’s test).

**Table 2:** Changes in measured outcomes between experimental and control groups.

Variable	Δ Experimental	Δ Control
Average skin temperature (°C)	−16.02 °C	+0.13 °C
Distal motor latency (median) (ms)	+0.05 ms	−0.05 ms
Motor NCV for median nerve (m/s)	−10.37 m/s	+0.33 m/s
Distal motor latency (ulnar) (ms)	−0.75 ms	−0.03 ms
Motor NCV for ulnar nerve (m/s)	−8.79 m/s	+0.25 m/s
Onset latency for median nerve (ms)	+0.24 ms	−0.20 ms
Sensory NCV for median nerve (m/s)	−5.20 m/s	+0.70 m/s
Onset latency for ulnar nerve (ms)	−0.53 ms	−0.07 ms
Sensory NCV for ulnar nerve (m/s)	−8.40 m/s	+0.03 m/s

## Data Availability

The data of this study can be made available by the first author upon reasonable request.

## Abbreviations

The following abbreviations are used in this manuscript:

APB	Abductor Pollicis Brevis
ADM	Abductor Digiti Minimi
BMI	Body Mass Index
CMAP	Compound Muscle Action Potential
DML	Distal Motor Latency
NCV	Nerve Conduction Velocity
ROI	Region of Interest
SD	Standard Deviation
